# An ecological paradox: high species diversity and low position of the upper forest line in the Andean Depression

**DOI:** 10.1002/ece3.1078

**Published:** 2014-04-30

**Authors:** Thorsten Peters, Achim Braeuning, Jannes Muenchow, Michael Richter

**Affiliations:** Institute of Geography, Friedrich-Alexander-University Erlangen-Nuremberg91054, Erlangen, Germany

**Keywords:** Andean Depression, Andes, climate, forest structure, plant diversity, tree diversity, upper forest line

## Abstract

Systematic investigations of the upper forest line (UFL) primarily concentrate on mid and high latitudes of the Northern Hemisphere, whereas studies of Neotropical UFLs are still fragmentary. This article outlines the extraordinary high tree diversity at the UFL within the Andean Depression and unravels the links between the comparatively low position of the local UFL, high tree-species diversity, and climate. On the basis of Gentry′s rapid inventory methodology for the tropics, vegetation sampling was conducted at 12 UFL sites, and local climate (temperature, wind, precipitation, and soil moisture) was investigated at six sites. Monotypic forests dominated by *Polylepis* were only found at the higher located margins of the Andean Depression while the lower situated core areas were characterized by a species-rich forest, which lacked the elsewhere dominant tree-species *Polylepis*. In total, a remarkably high tree-species number of 255 tree species of 40 different plant families was found. Beta-diversity was also high with more than two complete species turnovers. A non-linear relationship between the floristic similarity of the investigated study sites and elevation was detected. Temperatures at the investigated study sites clearly exceeded 5.5°C, the postulated threshold value for the upper tree growth limit in the tropics. Instead, quasi-permanent trade winds, high precipitation amounts, and high soil water contents affect the local position of the UFL in a negative way. Interestingly, most of the above-mentioned factors are also contributing to the high species richness. The result is a combination of a clearly marked upper forest line depression combined with an extraordinary forest line complexity, which was an almost unknown paradox.

## Introduction

Systematic investigations of the upper forest line (UFL) mainly concentrate on mid and high latitudes of the Northern Hemisphere, whereas studies of tropical UFLs are still fragmentary (e.g., Walter and Medina 1969; Ellenberg 1979; Miehe and Miehe 1996; Kessler and Hohnwald 1998; Körner 1998, 2007; Lauer et al. 2001a; Holtmeier 2003; Bader and Ruijten 2008). Along the north–south stretching tropical Andes, the factors that determine its altitudinal position are not well understood and little information is available about tree diversity and species distribution at the UFL. Since at least 7000 years, Andean landscapes have been modified by humans, and the poignancy of land use has grown during the past fifty years (Jokisch and Lair 2002; Sarmiento and Frolich 2002; Peters et al. 2010; Rodriguez et al. 2013). Although climate change is intensely debated as a cause of future species extinctions at UFLs, human land use is currently the most important threat to biodiversity in the tropics (Pimm and Raven 2000; Sala et al. 2000; Köster et al. 2009). As early as 1958, Ellenberg claimed isolated and insular *Polylepis* woodlands of the high Andean mountain areas to be relics of a former closed forest zone, which has been destroyed by anthropogenic fires and extensive pasture management (Ellenberg 1979). Several studies (e.g., Walter and Medina 1969; Miehe and Miehe 1996; Kessler and Hohnwald 1998; Körner 1998, 2007; Lauer et al. 2001b; Körner and Paulsen 2004) show that temperature is the most important natural factor for limiting tree growth. Körner (1998, 2007) and Körner and Paulsen (2004) demonstrate that soil temperatures >5.5°C (measured close to the rhizosphere 10 cm below ground surface) are essential for tree growth at the UFL ecotones in the tropics. On a local scale, further climate elements (e.g., wind, precipitation, soil moisture) and environmental factors such as relief form, different types of slope processes, and the occurrence of fires affect the position of the natural UFL as well (Holtmeier 2003; Tinner and Theurillat 2003; Holtmeier and Broll 2005; Bader et al. 2007a,b2007b; Treml and Banaš 2008).

Within the Andean chain, the relatively low-lying regions of the so-called Andean Depression located between northern Peru and southern Ecuador are of special interest since this area forms an important migration corridor for tree species of different plant geographical areas during the recent climate history (Richter et al. 2009). Studying tree-species diversity and climate at the UFL of the Andean Depression is central to understand tree-species distribution and forest structure patterns within this important transition zone between the central and northern Andes. The objectives of this study are to: (1) record the exceptionally high tree-species diversity at the UFL within the Andean Depression, (2) document changes in species composition, forest structure, and location of the local UFLs, and (3) unravel the links between the position of the UFL, tree-species diversity, and climate within the study area.

## Materials and Methods

### Study region

**Figure 1:**
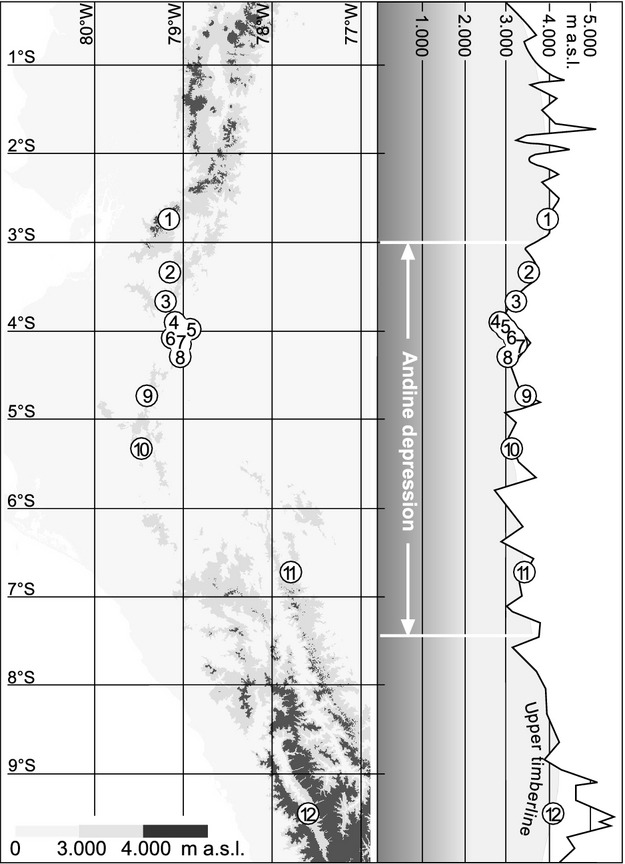
Location of the twelve investigated UFL study sites. Map based on the 2012 (SRTM) dataset 2012. For detailed information, refer to Table 1.

The study was carried out between El Cajas in southern Ecuador and Llanganuco in northern Peru (Fig. 1, Table 1). Except for El Cajas and Llanganuco, the investigated areas are part of the “Andean Depression” (also known as Depression of Huancabamba) which separates the central Andes from the northern Andes. This mountain section, where the highest peaks barely reach 4000 m a.s.l., stretches about 500 km north–south between the Girón-Paute drainage basin around Cuenca in southern Ecuador and the Rio Chicama-Rio Huallaga intersection around Cajamarca in northern Peru (Weigend 2002, 2004). Within the Andean Depression, the UFL is situated lower than in the northern and southern Andes. Here, the Ecuadorian Andes includes one of the world′s five hotspots of vascular plant diversity, where approximately half of the estimated 20,000 Ecuadorian vascular plant species occur (Balslev 1988; Jorgensen and León-Yánez 1999; Barthlott et al. 2007). The region's climate is dominated by the tropical trade wind system. Especially, high-elevation crest lines and adjacent upper forest line ecotones are affected by strong easterlies over 70% of the year. Maximum amounts of rainfall are measured at the eastern slopes of the Andean chain, where the predominant easterlies are lifted by forced convection (e.g., >6000 mm year^−1^ in several windward locations of the Andean Depression; Emck 2007). The specific study sites are located in the “tierra fria” (mean annual temperature ≈ 9–10°C at 3000 m a.s.l.) and “tierra subhelada” (mean annual temperature ≈ 6–7° C at 3500 m a.s.l.; Richter 2003) altitudinal belts. The topography of the central Andean Depression is structured by deep ravines, steep slopes, and narrow ridge tops (Richter et al. 2009).

**Table 1:** Site details for the 12 investigated UFL locations in southern Ecuador and northern Peru

No.	Study site (abbr.)	Coordinates	Mean altitude (m a.s.l.)	Climate measurement period
1	El Cajas (EC)	2°46′ S; 79°12′ W	∼3900	
2	Fierro Urco (FU)	3°43′ S; 79°19′ W	∼3530	22.05.2007–19.02.2008
3	Saraguro (Sa)	3°40′ S; 79°14′ W	∼3270	22.05.2007–19.02.2008
4	El Tiro (ET)	3°58′ S; 79°8′ W	∼2800	01.01.2005–28.02.2008
5	Cerro del Consuelo (CC)	3°59′ S; 79°3′ W	∼2890	01.01.2005–28.02.2008
6	Cajanuma (Ca)	4°7′ S; 79°9′ W	∼3240	01.01.2005–28.02.2008
7	Rabadilla de Vaca (Ra)	4°15′ S; 79°7′ W	∼3250	
8	Cerro Toledo (CT)	4°22′ S; 79°6′ W	∼3130	
9	Amaluza (Am)	4°44′ S; 79°25′ W	∼3530	30.04.2006–04.03.2007
10	Huancabamba (Hu)	5°20′ S; 79°32′ W	∼3190	
11	Barro Negro (BN)	6°42′ S; 77°54′ W	∼3550	
12	Llanganuco (Ll)	9°3′ S; 77°35′ W	∼4.250	

### Vegetation sampling

The selection of twelve UFL sites (Fig. 1, Table 1) was based on topographic maps (map scale 1:50,000) and aerial photographs of the “Instituto Geográfico Militar” of Ecuador. Based on Gentry′s rapid inventory methodology for the tropics (Gentry 1982, 1988), eight transects à 50 m × 2 m were selected at each study site and trees with a stem diameter >5 cm at 30 cm above ground level were identified. Due to the sometimes restricted size of forest areas and the complex topography of the region, Gentry′s method was modified by taking an irregular layout of the 2 × 50 m transects (transects parallel to equal altitude and without splitting the lines) and by investigating only eight instead of 10 plots at each study site. Collected specimens were identified in the herbaria of the Universities of Loja (Universidad Nacional), Cuenca (Universidad del Azuay), and Quito (Universidad Católica).

### Statistical analysis of vegetation data

In a first step, we used cluster analyses to compare the floristic composition of the different sampling plots. Secondly, detrended correspondence analysis was applied to identify relationships between species composition and environmental factors such as temperature, wind speed, precipitation, elevation, slope, aspect, latitude, and longitude. Thirdly, ordinations and modeling techniques were coupled to link the community patterns to environmental predictors (Muenchow et al. 2013a,b2013b). An initial linear model, trying to fit the scores of the first DCA-axis, revealed spatial autocorrelation, since the transects of each location were more similar to one another as to those from other locations. Therefore, we used a linear mixed-effects model in which location was treated as random intercept. A random intercept model allows for correlation within a class (here location), thus accounting for the observed spatial autocorrelation (Zuur et al. 2009). Moreover, to model the non-linear relationship between the first DCA-axis and elevation, elevation was subjected to an orthogonal polynomial function of the third order. Following a hypothesis-testing modeling selection procedure, we had to dismiss all further topographical predictors one at a time in accordance with an *F*- test (*P* value > 0.05). Further model inspection revealed no violations of model assumptions (normality, heterogeneity, independence). As a measure of fit, we used the coefficient of determination between the fitted values and the response. The intraclass correlation (correlation between the transects from the same location) was computed as the ratio of the variance of the random effects and the sum of the variance and the squared residuals of the random effects (Zuur et al. 2009). Additionally, we used species accumulation curves to decide whether our sampling approach provided a representative sample of the expected forest line tree species within our study area. We used the algorithm recommended by Oksanen (2013). It was developed independently by various authors (Ugland et al. 2003; Colwell et al. 2004; Kindt et al. 2006). For the statistical modeling and species accumulation curves, we used the open-source software R (R Development Core Team 2014) and its packages lattice (Sarkar 2008) lme4 (Bates et al. 2014) and vegan (Oksanen et al. 2014).

### Climate measurements

Local climate was recorded at six of the UFL sites (Table 1) by installing nine full automatic climate stations (manufactured by THIES Clima Germany or Hobo Onset USA) inside and outside of local forest stands (Peters 2009). Each climate variable was recorded in 10 minute intervals, and hourly arithmetic means (for wind direction vectorial mean) were stored by data loggers. At all sites, air temperatures were taken 2 m above ground level and soil temperatures at 10 cm ground depth. Wind velocities and directions were measured 2.5 m above ground. Wind as a decisive force for the position of the UFL was investigated in detail at Cajanuma Páramo, where wind direction and wind speed were studied at four sites at the crest line and at the lee side of the Cordillera de San Francisco. Precipitation and soil moisture were analyzed at Cajanuma in and outside local forest stands.

## Results

### Tree diversity and distribution

**Figure 2:**
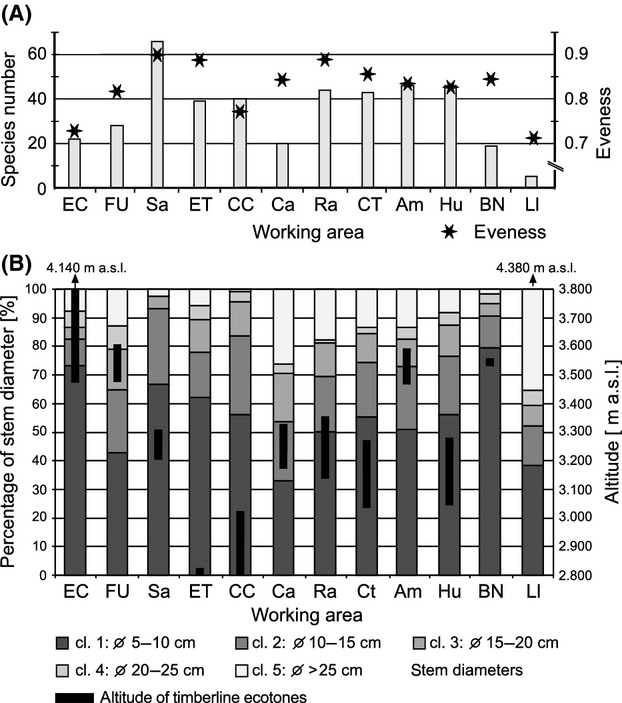
(A) Numbers of tree species and evenness values per study site and (B) classified stem diameters and altitudes of the local UFLs. For site codes, refer to Table 1.

**Figure 3:**
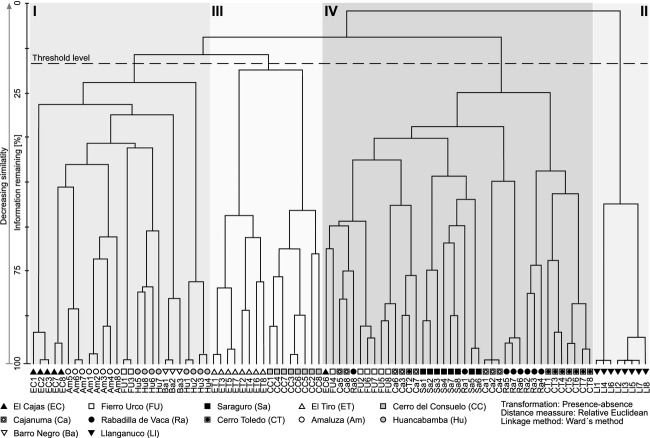
Classification of the sampled vegetation transects, showing the degree of dissimilarity in tree-species composition.

**Figure 4:**
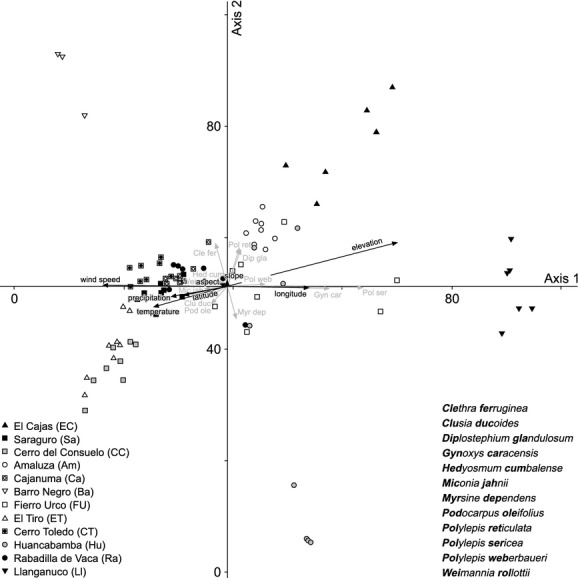
Detrended correspondence analysis (DCA) showing the floristic similarity of the investigated study sites. The most important indicator species as well as side factors were shown by gray and black arrows, respectively. Total variance 15.01; axis 1 eigenvalue = 0.87 (gradient length 4.344); axis 2 eigenvalue = 0.70 (gradient length 6.553).

**Figure 5:**
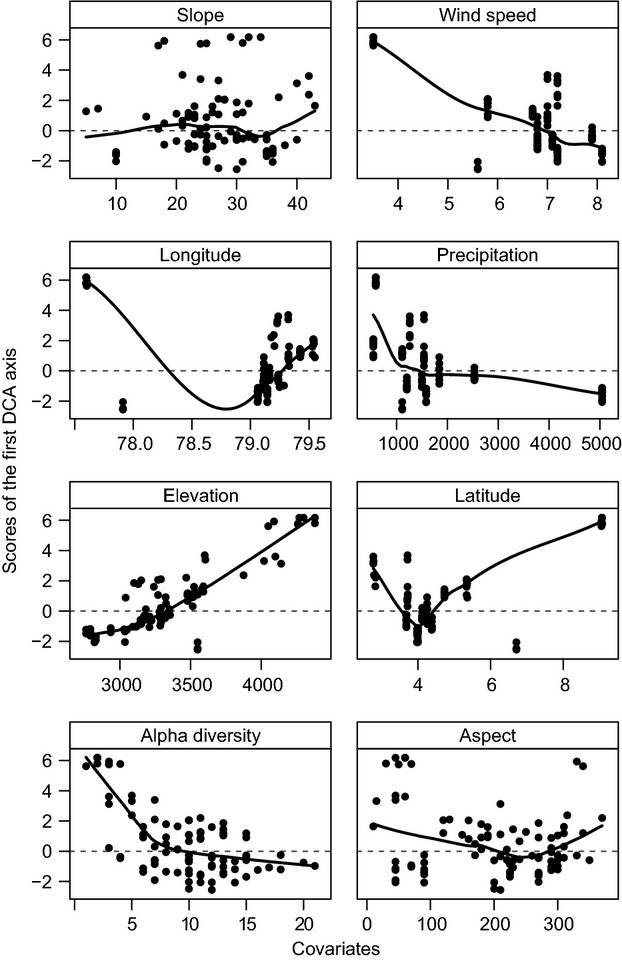
Scatter plot of available covariates versus the scores of the first DCA-axis. A line smoother was added to aid visual inspection. Units of the variables: scores of the first DCA-axis in SDs; aspect, latitude, longitude, and slope in °; elevation in m a.s.l.; precipitation in mm; wind speed in ms^−1^.

**Figure 6:**
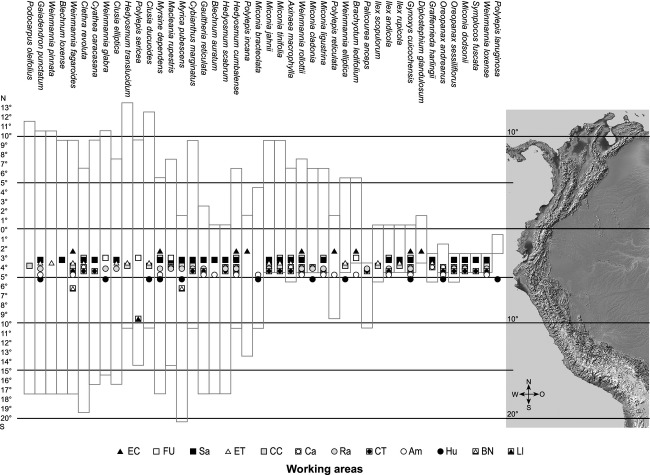
Distribution areas of the 45 most frequent tree species of the investigated UFLs. The bars show the distribution of the species as listed by Missouri Botanical Garden (2013).

Syntaxonomical tree studies at the UFL of the Andean Depression show remarkably high tree-species numbers (Fig. 2A). In total, 255 tree species (including 94 morpho species) of 40 different plant families were identified within 96 transects (e.g., a total area of only 9600 m^2^). Beta-diversity was high with more than two complete species turnovers (length of the first DCA-axis: 8.73). Using species accumulation curves (graph not shown), we found the expected number of species for the visited UFLs. The calculated species richness is 253.8 (Standard deviation: SD = 1.4), what means that the number of the tree species of the visited sites (255 tree species) matches almost entirely the expected mean diversity. Melastomataceae (45 spp.), Asteraceae (31 spp.), Araliaceae (15 spp.), and Cunoniaceae (15 spp.) are the most species-rich families, while Melastomataceae and Rosaceae are the most common, with 18% and 13% of all tree individuals, respectively. The highest quantity of 66 tree species per 800 m^2^ was sampled in the strongly anthropogenically affected study area of Saraguro, where forest stands are dominated up to 66% (Fig. 2A and B) by trees showing stem diameters of only 5–10 cm. Species richness and evenness are maximal in Saraguro, whereas the lowest species numbers were collected in El Cajas and Llanganuco, outside of the Andean Depression. We suggest that this low diversity is the consequence of the comparatively high altitude of the two study sites and the dominance of *Polylepis incana*, *P. sericea*, *P. reticulata,* and *P. weberbauerii* (Rosaceae) representing more than 60% of tree individuals at these sites. Within the Andean Depression species numbers and evenness values are clearly increased, varying from 0.8 to 0.9. One exception is the site Cajanuma, where a high proportion of trees with stem diameters of >25 cm induces a smaller number of tree individuals and thus of tree species. Statistical analyses on the species level reveal a conspicuous floristical division of the investigated study sites into four groups (I–IV, Fig. 3). Group I contains the working areas of El Cajas, Amaluza, Fierro Urco, and Huancabamba which are characterized by the indicator species *Polylepis reticulata* (*P* < 0.018), *P. weberbaueri* (*P* < 0.007; both Rosacea), *Myrsine dependens* (*P* < 0.002; Myrsinaceae)*, Diplostephium glandulosum* (*P* < 0.049; Asteraceae)*, Clethra ferruginea* (*P* < 0.048; Clethraceae), and *Miconia bracteolata* (*P* < 0.008; Miconiaceae) (Fig. 4). *Polylepis reticulata* and *P. weberbaueri* are very common within the higher situated UFLs of the north and central Andes, and numerous populations have been described for the higher regions of Ecuador and Peru (up to 4500 m a.s.l.; Jorgensen and León-Yánez 1999; Missouri Botanical Garden 2013). This holds also true for *Myrsine dependens*, *Diplostephium glandulosum,* and *Miconia bracteolata* which were found at altitudes up to 4500 m a.sl. (Jorgensen and León-Yánez 1999; Missouri Botanical Garden 2013). Group II consists of the eight transects at the Llanganuco study site. This group is distinguished by the occurrence of *Polylepis sericea* (*P* < 0.001), *P. weberbaueri* (both Rosaceae), and *Gynoxis caracensis* (*P* < 0.001; Asteraceae), representing characteristic tree species of the higher located ULFs of the Andes (Fig. 4, Baumann 1988). Group III contains the two lowest situated study areas El Tiro and Cerro del Consuelo. Both are floristically clearly different from the remaining areas (Fig. 3; threshold level 1). This is mainly due to the presence of *Podocarpus oleifolius* (*P* < 0.001; Podocarpaceae) and *Clusia ducoides* (*P* < 0.012; Clusiaceae) (Fig. 4). Both species are very common in the lower humid areas of the Neotropics, and distribution areas are described for Ecuador up to 3200 m a.s.l. (Smith et al. 2004; Missouri Botanical Garden 2013). Group IV is comprised of the remaining middle elevation study sites (Fig. 3). These relevés are characterized by the occurrence of *Weinmannia rollottii* (*P* < 0.001; Cunoniaceae), *Miconia jahnii* (*P* < 0.006; Melastomataceae), and *Hedyosmum cumbalense* (*P* < 0.004; Chloranthaceae) (Fig. 4). While the first two species were described for Ecuador up to 3500 m a.s.l., *Hedyosmum cumbalense* was found at even higher altitudes up to 4500 m a.s.l. (Missouri Botanical Garden 2013). There is a non-linear relationship between the scores of the first DCA-axis and elevation. Furthermore, transects of the same location show spatial autocorrelation. Our linear mixed-effects modeling approach in combination with a polynomial function provided the solutions to both challenges. The *R*^2^ between the fitted values and the response is 96%. The intraclass correlation is 0.92. As shown by Fig. 5, the scores of the first DCA-axis also show a strong correlation with decreasing amounts of wind speed, precipitation, and alpha diversity and no interrelations with slope, longitude, latitude, and aspect. Unfortunately, the same available topographic and climatic predictors show no significant relationship with the second DCA-axis. Concerning the distribution patterns of the 45 most frequent tree species, the major part has been found in this section of the Andes before (see bars Fig. 6). Only a few tree species (e.g., *Graffenrieda harlingii*, *Oreopanax sessiliflorus,* and *Weinmania loxense*) are restricted to the Andean Depression and have to be considered as endemic to the region. According to our study, the known distribution limits for some species (e.g., *Brachyotum ledifolium*, *Gynoxis cuicochensis*, *Ilex andicola*, *Miconia cladonia*, *Oreopanax andreanus*, *Polylepis lanuginose, P. reticulata*, *Weinmannia rollottii*, *W. elliptica*) must be expanded toward the south. None of the presented tree species was sampled within all investigated study sites, and most species show very heterogeneous distribution patterns (Fig. 6).

### Climate

#### Temperature

Average annual air temperatures at the UFL sites range from 5.6°C at Amaluza to 9.6°C at Cerro del Consuelo. Soil temperatures are even higher, ranging between 8.9°C at Amaluza to 11.4°C at Cerro del Consuelo. Temperature variations (expressed by standard deviation SD) are highest at Cerro del Consuelo for soil temperatures (SD 2.3) and at Saraguro and Amaluza for air temperatures (SD 1.9) (Table 2). Differences between soil and air temperatures were generally larger at the higher study sites. Diurnal air temperature amplitudes are largest at the sites of Fierro Urco, Cajanuma Páramo, and Amaluza, varying from 3.2–5.1 K. In comparison, soil temperature amplitudes only vary between 0.7 K at Amaluza to 2.1 K at Cajanuma Páramo and Fierro Urco. Absolute minimum temperatures of 0.8°C were measured at Cajanuma Páramo and Fierro Urco. Mean daily temperatures below 5.5°C, which corresponds to the postulated upper threshold for tree growth in the tropics (Körner and Paulsen 2004; Körner 2007), are reflected in low frequencies. Only at Amaluza and Fierro Urco, frequencies of 59.35% and 32.78% hint to a limitation of tree growth possibly driven by temperature. Both, air as well as soil temperatures vary during the year, since seasonal minimum temperatures come along with a perhumid period between May and August.

**Table 2:** Comparison of air (+2 m) and soil temperatures (−10 cm) at five UFLs

Area Recording period	Type of data	Tmean (SD) (°C)	Tmin (°C)	Tmax (°C)	Mean daily amplitude (K)	Percentage of T ≤ 5.5°C
Fierro Urco 23.5.07–18.2.08	Tair (+2 m)	6.4 (1.7)	1.6	13.7	3.7	32.78
	Tsoil (−10 cm)	9.1 (2.0)	4.4	15.6	2.1	0.94
Saraguro 23.5.07–18.2.08	Tair (+2 m)	7.4 (1.9)	5.9	16.7	2.4	0
	Tsoil (−10 cm)	11.3 (1.7)	2.9	14.9	3.7	9.73
Cajanuma Páramo 1.1.98–17.9.06	Tair (+2 m)	6.8 (1.7)	0.8	18.6	3.2	18.6
	Tsoil (−10 cm)	10.5 (1.6)	3.8	19.4	2.1	0.18
Cerro de Consuelo 1.1.98–17.9.06	Tair (+2 m)	9.6 (1.6)	3.7	20.9	2.9	0.13
	Tsoil (−10 cm)	11.4 (2.3)	4.2	23.2	4.2	0.03
Amaluza 7.10.06–3.3.07	Tair (+2 m)	5.6 (1.9)	0.8	16.3	5.1	59.35
	Tsoil (−10 cm)	8.9 (0.9)	5	10.6	0.7	0.76

#### Wind

**Figure 7:**
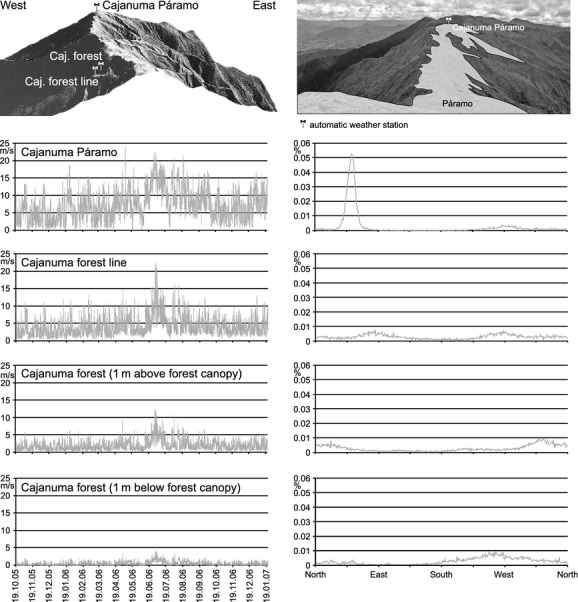
Wind speeds (left panel) and wind directions (right panel) inside and outside of local forest stands at Cajanuma. Photo: Peters 2013.

Mean wind speed ranges between 7.1 ms^−1^ at El Tiro and Cajanuma Páramo to 2.4 ms^−1^ at Amaluza. Maximum wind speeds were recorded at Cajanuma Páramo (24.0 ms^−1^) and El Tiro (20.5 ms^−1^) where winds show an impressive directional perseverance driven by easterlies (Table 3). Storms (>16.9 ms^−1^) occur all over the year with highest frequencies of 2.25% at the study site of Cajanuma. Comparatively, calms are rare, showing values from 2.11% at Amaluza to 0% at Cajanuma Páramo. Wind as an important force for determining the local position of the UFL was investigated in detail at Cajanuma Páramo (Fig. 7). Mean wind speeds are highest on the crest itself (average wind speed 7.1 ms^−1^ at 3400 m a.s.l.). Only 150 m beyond the ridge, at Cajanuma forest line in 3235 m a.s.l., mean wind velocity is reduced to 4.1 ms^−1^) and even to 2.0 ms^−1^ within the forest at Cajanuma (1 m above the closed forest canopy at 3215 m a.s.l.). Lowest average wind speeds of only 0.5 ms^−1^ occur within Cajanuma forest 1 m below closed forest canopy. The easterlies dominating at the crest line are strongly dampened at the lee side. Here, wind fields are more balanced showing slight tendencies toward a mountain-valley wind system.

**Table 3:** Wind velocities and averaged wind directions at five UFLs

Area Recording period	Wind speed mean (m s^−1^)	Wind speed max (m s^−1^)	Easterlies (%)	Storms (%)	Calms (%)
Fierro Urco 22.5.07–19.2.08	6.3	13.7	n.a.	0.00	0.03
El Tiro 17.11.05–17.12.06	7.1	20.5	78.16	1.41	0.04
Cajanuma Páramo 19.10.05–19.1.07	7.1	24.0	64.69	2.25	0.00
Cerro de Consuelo 19.10.05–17.9.06	5.7	15.9	79.34	0.00	0.34
Amaluza 30.4.06–4.3.07	2.4	16.2	31.93	0.05	2.11

#### Precipitation and soil moisture

**Figure 8:**
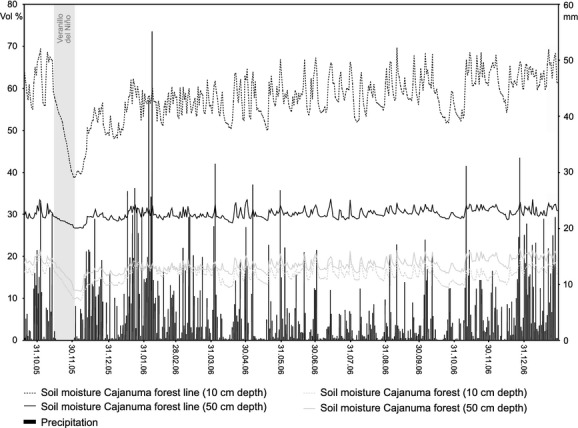
Daily precipitation amounts and soil moisture contents inside and outside of local forest stands at Cajanuma.

Rainfall observations along the Andes of southern Ecuador and northern Peru present extraordinary high amounts of rainfall at the northern part of the Andean Depression (Killeen et al. 2007; Peters 2009). Here, more than 6000 mma^−1^ are registered at Lagunas Compadres close to Cajanuma (Emck 2007). The resulting high soil moisture levels at Cajanuma indicate significantly different soil water contents between local forest stands and open páramo sites (Fig. 8). Soils of the open grass and scrubland develop on solid rocky layers, which cause long-lasting water stagnation, causing considerably higher soil moisture content than in the coarse porous debris layer beyond the forests. However, thick isolating organic layers in elfin forests guarantee that these soils never dry up completely. Elfin forests create their own water regime by a thick organic stratum that conserves moisture also during rarely occurring events of several weeks lasting dry periods during “Veranillo del Niño” from October to November, e.g., given during the second half of November 2005.

## Discussion

*Polylepis* sometimes joined by *Gynoxis* is the most important indicator tree species of the higher situated UFLs outside of the Andean Depression. This is best demonstrated by comparing two UFL sites from northern Ecuador (Páramo de Papallacta) and central Peru (Cordillera Blanca). At Papallacta, the UFL is situated between 3700 m and 4700 m a.sl. and *Polylepis pauta*, *P. incana*, *Gynoxis buxifolia*, *G. acostea,* and *Escallonia myrtilloides* are the prevailing tree species (Lauer et al. 2001b, 2003). South of the Andean Depression, the UFL of the Cordillera Blanca is located at almost the same altitude between 3800 m to 4550 m a.s.l. Here, *Polylepis weberbauerii*, *P. sericea*, *Gynoxis caracensis,* and *Buddleja usush* are dominant (Kull 1991). At the study sites within the Andean Depression, monotypic forests dominated by *Polylepis* were only found at El Cajas, Fierro Urco, Huancabamba, Barro Negro, and Llanganuco, all located at the margins of the Andean Depression. In contrast, the core areas are characterized by a species-rich forest, which lacks the elsewhere dominant UFL genus *Polylepis*. Strong winds and high precipitation amounts are considered to be the most important reasons for the lack of *Polylepis* in the northern section of the Andean Depression, because the genus tends to avoid perhumid regions (Kessler 1995; Richter and Moreira-Munoz 2005; Brunschön and Behling 2010). One of the most surprising results is the high number of 255 tree species (including 94 morpho species) of 40 different plant families at the UFLs of the study area. On global scale, high species numbers result from diversity triggers such as the so-called “Rapoport′s rule” (Willig et al. 2003) and the effective evolutionary time since the mid-tertiary (Rohde 1992; Beck and Richter 2008). On regional scale, the northern Andes form an important interface between the Tumbes-Chocó-Magdalena hotspot in the west and the Amazonian lowlands in the east (Richter et al. 2009). On a local scale, the tropical section of the Andean mountain chain is characterized by various taxa of constrictive range. Here, harsh climatic conditions (e.g., high wind speeds and precipitation amounts) as well as a complex topography cause a manifold pattern of tree species. Besides natural factors, also anthropogenic disturbances have to be considered as triggers for the area′s outstanding plant diversity. Especially at the ULFs in the vicinity of Saraguro, including Cerro Toledo, Amaluza, and Huancabamba, human influence is clearly visible. Here, the UFL is composed of small forest patches and the forest-páramo border is always sharp. A relatively high amount of trees showing a stem diameter of < 15 cm induces a higher number of tree individuals and thus of tree species. These forests resemble secondary stands, including many treelets of “pioneer” character with softwood and large hydromorphous leaves. Soil temperatures as well as mean air temperatures at these UFLs clearly exceed 5.5°C, the postulated threshold value for the upper tree growth limit in the tropics (Körner 1998, 2007; Körner and Paulsen 2004). Consequently, temperature cannot be considered as the main cause for the low position of the local UFL ecotone. Having regard to the floristic similarity of the investigated study sites, elevation almost completely explained the first DCA-axis of the presented detrended correspondence analysis. Elevation also represents closely correlated ecological parameters such as temperature (Emck 2007), precipitation (Fries et al. 2012; Muenchow et al. 2013c), wind speed (Richter et al. 2008; Peters 2009), and/or soil conditions (Muenchow et al. 2013a). In our study area, quasi-permanent trade winds and extraordinary high precipitation amounts as well as high soil water contents are affecting the local position of the UFL in a negative way. Especially, the strong easterlies constrain the establishment of forests at the upper ridge areas of the Páramo formations. The consequence is a patchy structure of isolated but dense tree stands in shallow depressions or on wind-protected flats on the windward escarpment below the crest line. In contrast, dense forests occur just beneath the rim of the same crest forming a sharp forest line on the leeside. In addition, high precipitation amounts are leading to higher soil water contents on the ridges, thus inhibiting tree growth. Depending on topography, this phenomenon may already begin as low as 2600 m a.s.l. At lower elevations, the ridges disappear under a coherent forest cover as they become steeper and thus water drainage is more efficient. Since elevation also explains species richness pretty well (*R*^2^: 72%, model not shown), the first DCA-axis also constitutes a species richness gradient. Of course, increasingly harsh conditions at higher altitudinal levels affect adversely community composition and diversity. Some of the challenges plants encounter with increasing altitude are lower temperatures (Körner 1998; Bendix and Rafiqpoor 2001; Körner and Paulsen 2004), higher wind speeds (Holtmeier 2003; Peters 2009) and higher precipitation amounts (Emck 2007; Bendix et al. 2009), to name but a few. Interestingly, most of the above-mentioned climate factors are also contributing to the high species richness. On the one hand, high tree diversity can be explained by harsh environmental conditions as well as by a complex topography and also by the rain-induced poor soil conditions. On the other hand, the same factors are also responsible for the lowering of the UFL. The result is a combination of a clearly marked UFL depression combined with an extraordinary UFL complexity, which was an almost unknown paradox.
